# Building block for success: A case study of capacity-strengthening in grant administration for Pakistani universities

**DOI:** 10.1371/journal.pone.0314141

**Published:** 2024-11-22

**Authors:** Mustafa Hassan, Bakhtawar Ghafoor, Gerald Bloomfield, Ayeesha Kamal, Aysha Almas, Safia Awan, Muhammed Tariq, Salim Virani, Zainab Samad

**Affiliations:** 1 Department of Shared Research Services, The Aga Khan University, Karachi, Pakistan; 2 Duke Global Health Institute, Durham, North Carolina, United States of America; 3 Department of Medicine, The Aga Khan University, Karachi, Pakistan; The Aga Khan University Hospital (AKUH), PAKISTAN

## Abstract

**Background:**

Grant administration plays a pivotal role in the success of research and academic endeavors. However, in Pakistan, academic institutions often face challenges in this domain, primarily due to a lack of specialized training and infrastructure.

**Aims:**

This research paper presents the outcomes of a comprehensive capacity-strengthening workshop, hosted in two cities, aimed at improving grant administration skills in Pakistani academic institutions.

**Methodology:**

A pre and post-survey analysis was conducted to assess participants’ knowledge and experience. A pre-survey method was used to develop the learning objectives and content for the workshop, whereas the post-survey tool was used to assess the immediate effect of a two-day capacity-strengthening workshop on participants’ knowledge of grants

**Results:**

Response rates for Karachi participants were 86% pre and 87% post, while for Islamabad participants, they were 63% pre and 57% post. Notably, participants in Islamabad, where infrastructure and support services are often less advanced, exhibited significant improvement in their understanding of grant administration processes. Factors contributing to the workshop’s success included its dynamic content, interactive learning methods, and an inclusive learning environment.

**Conclusion:**

The outcomes of this research provide insights into the effectiveness of tailored capacity-strengthening programs in addressing the unique needs of diverse academic institutions. In addition, it highlights the potential of collaborative learning, where participants from various backgrounds come together to enhance their collective knowledge. This initiative underscores the importance of enhancing grant administration skills to foster a more robust research ecosystem in Pakistan.

## Introduction

Pakistan is home to a cadre of dedicated medical professionals grappling with the formidable burden of disease in a population of over 225 million people [1]. The state of healthcare in Pakistan is marked by a pressing need for transformative interventions, shaped by sound research and biomedical innovation. Such research plays a pivotal role in understanding the intricacies of local health challenges and devising context-specific solutions [1, 2].

Biomedical research serves as the cornerstone of medical progress, providing the foundation for innovations in treatments, diagnostic methods, and healthcare strategies. However, the transformative potential of research can only be realized with adequate funding [3]. In an ever-evolving landscape of health challenges, research is vital for scientific progress, enabling the development of novel solutions to improve millions of lives [4–6].

Grant administration is a comprehensive process that involves managing research funding from the pre-award phase, through post-award management, to project close-out. The skill set required for effective grant administration includes proficiency in sourcing funding opportunities, understanding the legal and financial aspects of awards, developing and managing budgets, and ensuring compliance with grant requirements.

Effective grant administrators must also be skilled in managing the entire lifecycle of a grant, which involves tasks such as proposal development, budgeting, award negotiation, financial tracking, and reporting. In institutions with well-developed research infrastructures, like Aga Khan University (AKU), grant administrators play a crucial role in supporting principal investigators (PIs) and ensuring that the administrative aspects of research projects run smoothly. In contrast, many institutions in Pakistan lack dedicated personnel with these specialized skills, which makes building capacity in grant administration essential to improving the research ecosystem.

Grant administration plays a pivotal role even prior to securing funding, encompassing tasks such as identifying funding opportunities and providing essential support to Principal Investigators (PIs) in budget development. This early involvement is critical as PIs often encounter challenges in crafting budgets that align with grant requirements and project objectives. Additionally, Once researchers secure adequate funding to support their scientific pursuits, they face a formidable hurdle in the effective management and utilization of these funds [7, 8]. The landscape of grant management and administration in Pakistan is marked by several critical issues: inadequate infrastructure to support grant management, a shortage of skilled professionals, a lack of formalized training programs, and a dearth of specialized support functions such as dedicated finance, human resources, and research office infrastructure [9, 10]. These challenges can undermine the success and impact of research projects, hindering the achievement of their full potential [7, 11, 12].

The gaping void in knowledge and resources becomes increasingly evident when we examine academic institutions in LMICs with substantial research portfolios. While these institutions are prolific in generating research output, they often grapple with a crucial deficiency—a lack of comprehensive research management systems. This deficiency translates into a lack of trained personnel, and consequently, difficulties for principal investigators (PIs) who are forced to manage their grants without proper infrastructure and support. This not only jeopardizes the efficient utilization of grant funds but also undermines the credibility of research endeavors [10, 12, 13]. While some institutions, like Aga Khan University (AKU), have developed relatively strong research infrastructures, many others face significant challenges. Thus, capacity strengthening across institutions is essential to foster a more cohesive and effective research ecosystem.

In response to these pressing challenges in grant management within the academic landscape of Pakistan, our initiative sought to address both existing strengths and gaps. While some institutions, like Aga Khan University (AKU), possess relatively strong infrastructures and grant administration processes, many others, particularly in Islamabad, still face significant challenges. Thus, the workshop was designed with a dual purpose: to strengthen the existing capacities at institutions like AKU and to build capacity where it was lacking, particularly in institutions that have not yet developed robust systems for grant administration. Recognizing the urgent need for capacity-strengthening and effective training in grants management, we conducted the workshop as part of the National Center for Disease Research (NCD) Training Program, which was funded by the U.S. National Institutes of Health (NIH). To address the challenges of administering a research grant, a two-day workshop was designed specifically for Grant Administrators (GAs) and Principal Investigators (PIs). This workshop aimed to improve knowledge and skills in grant management among researchers and research administration staff.

## Methodology

### Study design

A Quasi-experimental approach was used to determine the needs and expectations of the participants [14]. A pre-and post-survey approach was employed [15]. A pre-survey method was used to develop the learning objectives and content for the workshop, whereas, the post-survey tool was used to assess the immediate effect of a two-day capacity-strengthening workshop on participants’ knowledge of grants administration.

### Need assessment

Initially, an open discussion with GAs and PIs from Aga Khan University was made to gather insights into their expectations and needs for capacity strengthening, revealing themes such as lack of understanding in pre-award, award, and post-award processes, as well as challenges in comprehending Funding Opportunity Announcements (FOAs), inexperience in budget development, award negotiation, post-award management, and project management. Later, based on the themes generated from general discussion with the GAs and PIs, a pre-workshop survey was developed which was distributed to all the participants via Google Forms to further get an insight into the expectations of the participants (pre-survey form is attached in appendix A).

Selection criteria for participants included early career research staff and faculty, gender balance, career aspirations, and diversity.

### Study population /setting

Two venues were chosen for the workshop: one in Karachi, primarily attended by grant administrators and early career researchers at Aga Khan University, and another in Islamabad, where the majority were principal investigators and early career researchers from 20 other research institutes across Pakistan. These institutions differ significantly in terms of research portfolio and infrastructure.

At AKU, the research infrastructure is well-established, supported by a dedicated team of grant administrators, finance officers, and compliance specialists who manage a broad research portfolio. This robust system ensures that researchers receive support at every stage of the grant lifecycle—from identifying funding opportunities to post-award management. In contrast, the institutions in Islamabad generally lack a similar level of specialized support. While these institutions may have research programs, they often lack dedicated teams for grant administration, and the researchers themselves are frequently responsible for managing both the scientific and administrative aspects of their grants. As a result, these institutions face challenges in navigating the complexities of grant administration, making the workshop’s capacity-building component crucial.

Selection criteria for participants included early career research staff, faculty, and individuals who may support grant management tasks. Although data collectors and research assistants are not typically responsible for managing grants, they often assist Principal Investigators (PIs) in administrative tasks such as budget tracking and documentation. Including these individuals in the workshop was important, as many expressed a desire to transition into formal grant administration roles as part of their long-term career development. By participating, they were able to gain foundational skills in grant administration, preparing them for future roles in research support.

### Development of training content

The workshop spanned two full days. We utilized a multimodal approach involving lectures, slide presentations, hands-on activities, group discussions, and active participant engagement through questions and discussions. All subject matter experts (SMEs) contributing to the workshop content possessed extensive expertise in grants management, with more than 10 years of experience in their respective fields.

### Key earning objectives

The learning objectives for the "Nuts and Bolts of Research Administration" workshop were carefully crafted based on a meticulous process that involved a comprehensive analysis of pre-survey outcomes and internal discussions with experienced Grant Administrators (GAs). This approach was designed to ensure that the workshop content would directly address the specific needs and expectations of the participants, creating a tailored learning experience.

The eight key learning objectives emerged from this process and were designed to encompass the essential components of grant administration. They include:

#### Develop proficiency in pre-award processes

Understanding the critical steps involved in preparing for grant applications, including identifying opportunities and understanding pre-award processes.

#### Develop proficiency in award processes

Focusing on the complexities of managing awarded grants, complexities of managing awarded grants, including different types of awards, contracts, and how to effectively read and interpret contracts.

#### Develop proficiency in post-award processes

Addressing the key responsibilities in managing a grant after it has been awarded, such as budget tracking and compliance.

#### Develop proficiency in close-out processes

Emphasizing the importance of completing all tasks and reports at the end of a project to ensure a successful close-out.

#### Learn basics of proposal development

Exploring the various phases involved in crafting a compelling grant proposal, from initial concept development to final submission.

#### Learn basics of budget development

Gaining insights into creating effective budgets for grant proposals and allocating funds for various project components.

#### Understand essential documentation requirements across the grant lifecycle

Navigating the paperwork involved in grant administration, from initial proposal submission to post-award documentation.

#### Gain proficiency in overall monitoring of grant-funded activities

Developing the skills necessary to oversee and manage grant-funded projects throughout their lifecycle.

These learning objectives were instrumental in shaping the content and structure of the workshop, ensuring that it provided a comprehensive and tailored training experience that met the specific needs of participants.

### Workshop content

The workshop content as shown in Table 1, was meticulously designed to encompass crucial aspects of grant administration based on the learning objectives described above.

**Table 1:** Workshop agenda: Topics covered in day 1 and day 2.

S#	Day 1	Day 2
**01**	Overview and Importance of Research	Post-award Management
**02**	Pre-award Management	Roles and responsibilities of PI and GA
**03**	Sourcing funding opportunities	Close-out Management
**04**	Stakeholder Engagement in Pre-award	Ethics in Research
**05**	Budget Preparation	Project Management Skills
**06**	Award	Career Development in Research Administration

Moreover, to engage the participants, on day 1, an interactive simulation game was used to reinforce learning and simulate real-world grant application scenarios. Participants engaged in tasks such as examining Funding Opportunity Announcements (FOAs), noting submission deadlines, identifying required documents, aligning project ideas with FOA objectives, understanding submission procedures, and practicing budget preparation. Through this hands-on exercise, participants gained practical experience and skills essential for navigating grant application processes effectively.

### Evaluation

The data collected from the post-survey questionnaires was analysed to assess the impact of the workshop on participants’ knowledge and satisfaction.

#### Post-survey questionnaire

Upon the completion of the workshop, a post-survey questionnaire was administered to assess the immediate impact of the training on their knowledge. The post-survey questionnaire is added to Appendix C and consists of questions rating various aspects of the training, participants’ confidence levels in different stages of grant administration, their confidence in specific topics, and their expectations regarding the impact of the training on their roles. Participants were asked to rate themselves on a scale of 1 to 5, with 1 indicating ’not confident at all’ and 5 indicating ’very confident.

### Statistical analysis

For statistical analysis, a paired t-test was used to compare the improvement in knowledge and understanding before and after training. Data were analyzed using SPSS version 26.

## Results

### Workshop setting

The workshop was conducted in Karachi and Islamabad, catering to a diverse group of participants from various departments and institutions. The geographical variation in the workshop settings provided a comprehensive understanding of the workshop’s impact across different backgrounds. The details of the workshop details are given in the Table 2.

**Table 2:** Demographic details of the workshop participants n = 64.

Details of the Participants of the Karachi Workshop				
Total Applications Received	Participants Selected	Departments Represented	Job Titles	Experience in Grant Administration
125	36	18	Data Collectors Research Associates Senior Research Assistants Assistant Managers Managers	1–4 Years
**Details of the Participants of Islamabad Workshop**				
170	38	Multiple Medical Institutes	Doctors Research Associates Assistant Professors Instructors Research Managers	1–4 Years

### Workshop content

#### Pre/post analysis of key learning objectives

To assess the workshop’s impact, a post-workshop evaluation was conducted. Participants were asked to rate their knowledge or familiarity with key grant administration processes before and after the workshop. Later, the results of post-workshop were compared with the pre-workshop to assess the workshop impact as shown in Table 3. The post-workshop analysis of key learning objectives in the Karachi and Islamabad workshops reveals substantial improvements in participant proficiency across all learning objectives.

**Table 3:** Pre/post analysis of key learning objectives.

	Score of Karachi Participants				Score of Islamabad Participants			
Learning Objectives	Pre-training score	Post-training score	% change	p value	Pre-training score	Post-training score	% change	p value
Develop proficiency in Pre-award processes	2.7	4.0	48%	<0.001	2.5	4.1	64%	<0.001
Develop proficiency in Award processes	2.5	3.8	52%	<0.001	2.5	4.0	60%	<0.001
Develop proficiency in Post-award processes	2.5	3.7	48%	<0.001	2.4	4.0	67%	<0.001
Develop proficiency in Close-out processes	2.4	3.7	54%	<0.001	2.2	3.8	73%	<0.001
Learn basics of proposal development	2.9	3.9	34%	<0.001	2.5	4.1	64%	<0.001
Learn basics of budget development	2.7	3.7	37%	<0.001	2.4	3.8	58%	<0.001
Understand essential documentation requirements across grant lifecycle	2.7	3.7	37%	<0.001	2.4	3.9	63%	<0.001
Overall monitoring of grant funded activities	2.6	3.7	42%	<0.001	2.4	3.9	63%	<0.001

#### Participants feedback and workshop ratings

In this section, we present the feedback and ratings provided by participants regarding the "Nuts and Bolts of Research Administration" workshop. Participants were asked to rate various aspects of the training on a scale of 1 to 5, with 1 indicating "Strongly Disagree" and 5 indicating "Strongly Agree." The following aspects were evaluated (Table 4):

**Table 4:** Participant feedback and workshop ratings (Percentage responses).

	Content was relevant to my role	Training was well-organized and structured	Sessions were engaging and interactive	Trainer has subject command	Duration was appropriate for covering the material
**Strongly Agreed**	38%	65%	60%	65%	58%
**Agreed**	56%	33%	31%	29%	31%
**Neutral**	4%	0%	7%	4%	7%
**Disagreed**	0%	0%	0%	0%	2%
**Strongly Disagreed**	2%	2%	2%	2%	2%

## Discussion

The main aim of this quasi-experimental evaluation was to investigate the effect of capacity-strengthening training intervention on the knowledge and awareness of participants about research grant administration. Moreover, this initiative underscores the importance of strengthening existing capacities in institutions with established infrastructure, while also addressing gaps in less developed institutions, to foster a more robust research ecosystem in Pakistan.

The effectiveness of the workshop can be attributed to its holistic approach to addressing key areas of grant administration. This emphasis was pivotal in driving the remarkable improvements observed in the participants.

One significant area of improvement was in the participants’ proficiency in pre-award processes. Pre-award activities represent a fundamental component of the grant lifecycle [8, 16, 17], and the substantial increase in participant proficiency in this area highlights the workshop’s efficacy in enhancing their understanding of these critical steps. The comprehensive content and the incorporation of hands-on activities allowed participants to grasp the intricacies of pre-award processes effectively.

The workshop’s focus on award management, a complex facet of grants, further contributed to the overall enhancement of participant knowledge and skills. Award management requires a deep understanding of legal terms and conditions of the contract being shared by the agency stating the crucial aspect of grant such as finances, project timelines, deliverables, and reporting mechanisms. Moreover, negotiations with the funder is the fundamental activity of this stage [17, 18]. The workshop’s in-depth coverage of these topics equipped participants with the necessary expertise.

Additionally, the post-award processes and close-out procedures also witnessed remarkable improvements. These aspects are often overlooked in grant administration despite their critical importance in the efficient execution and completion of research projects [17, 19–21]. The workshop’s detailed and practical exploration of these topics empowered participants to effectively navigate these crucial phases, ensuring better project outcomes and adherence to grant requirements.

Furthermore, the success of the workshop can be attributed to its participant-centric approach. It addressed the specific needs and knowledge gaps of the attendees, making the content highly relevant to their roles. This tailored approach fostered a more engaging and interactive learning environment, further contributing to the substantial improvement in participants’ grant administration skills.

In 2012, Temples et al. [22] conducted a pre- and post-training survey among departmental research administrators at the University of Central Arkansas to assess the immediate impact of research grant administration training. Their findings revealed that over 70% of participants expressed a significant positive response, indicating that the workshop effectively enhanced their understanding of research administration fundamentals and addressed specific challenges they encountered.

### Factors influencing the improvements

The substantial improvement observed in both locations can be attributed to the workshop’s comprehensive content and interactive teaching methods. The workshop’s focus on key aspects of grant administration, such as pre-award processes, award management, post-award processes, and close-out procedures, contributed to the participants’ enhanced understanding. Moreover, the incorporation of hands-on activities, group discussions, and interactive sessions created an engaging learning environment [23–25].

### Comparative analysis

The significant difference in improvement rates between Karachi and Islamabad participants may be linked to variations in infrastructural support across their respective institutions. Aga Khan University (AKU) in Karachi, known for its robust research infrastructure and well-established grant administration systems, provided a conducive environment for learning. The higher baseline knowledge in Karachi participants, as reflected in their pre-training scores, may be attributed to the institution’s existing support structures.

Participants from other institutions who attended the workshop in Islamabad, while facing differences in infrastructural support compared to AKU, showed a greater percentage of improvement. The workshop effectively addressed critical knowledge gaps for these participants, equipping them with essential skills for effective grant administration. Positive feedback about the workshop’s content, organization, and trainer expertise from participants underscores the program’s success.

### Participants’ feedback and workshop ratings

The results reflected the overall success of the workshop in meeting participants’ expectations and delivering a valuable learning experience. The high ratings in content relevance, organization, engagement, and trainer expertise affirm the workshop’s effectiveness in providing relevant and engaging training in research administration.

#### Content relevance

Participants’ feedback indicated a high level of satisfaction with the content’s relevance to their roles. The average rating for content relevance was 20, with 94% of participants rating it 4 or higher, indicating that the workshop effectively addressed their professional needs.

#### Training organization

The organization and structure of the training received positive feedback, with an average rating of 20. The majority of participants (98%) rated the training organization as 4 or higher, highlighting its effectiveness in providing a well-structured learning experience.

#### Engagement and interactivity

Participants found the sessions to be engaging and interactive, as reflected in an average rating of 20. 91% of participants rated the sessions as 4 or higher, indicating that the workshop successfully fostered an interactive learning environment.

#### Trainer expertise

The trainer’s subject command was a significant strength of the workshop, with an average rating of 20. A substantial percentage of participants (94%) rated the trainer’s expertise as 4 or higher, underscoring the effectiveness of the trainer in delivering the content.

#### Duration appropriateness

The duration of the training received positive feedback, with an average rating of 20. A majority of participants (89%) rated the training duration as 4 or higher, indicating that it was appropriate for covering the material effectively.

Our findings align with those of Temples et al. [22] who emphasized the significant utility of such training programs for university grant administrators. Moreover, beyond administrators, faculty members should actively participate in these sessions to enhance their understanding of grant administration, thereby facilitating more effective project execution. Temples et al. further recommended extending both the duration and frequency of these workshops, while also advocating for deeper, more comprehensive content to cater to advanced levels of training.

Following the discussion of the relevance of training programs for grant administrators and faculty members, it’s important to acknowledge the limitations of our study. Firstly, our sample selection may not fully represent the diverse landscape of grant administration needs across Pakistan, potentially limiting the generalizability of our findings. Additionally, while improvements in knowledge in post-survey are promising, it’s essential to recognize that enhanced self-efficacy may not necessarily translate to improved performance in grant administration practices.

While the workshop effectively enhanced participants’ knowledge and skills in key aspects of grant administration such as pre-award processes, award management, post-award processes, and close-out procedures, it is important to recognize that these are intermediate outcomes. The long-term effectiveness of the workshop in facilitating smoother research processes for Principal Investigators (PIs) will require longitudinal evaluation. A change in knowledge does not automatically translate into improved practices. Therefore, future studies should focus on evaluating how well the skills learned during the workshop are applied in practice and how they contribute to more efficient grant management and research processes over time.

Furthermore, While the workshop successfully enhanced participants’ knowledge and skills in grant administration, further steps are necessary to ensure that these gains translate into sustained improvements over time. Potential avenues for building on the skills acquired include offering follow-up workshops, introducing mentorship programs, exploring certification options, and fostering collaboration through shared knowledge events. These initiatives, if implemented, could provide ongoing support for participants and help further strengthen grant administration capacity across institutions in Pakistan.

These limitations underscore the need for future monitoring of quality improvement of the grants administration process and research to address these challenges comprehensively and further validate the effectiveness of capacity-strengthening initiatives in this field.

## Future directions

Recognizing the significant impact of the capacity-strengthening workshop on participants’ grant administration skills and understanding, it becomes evident that there is a pressing need for further advancements in this field. The workshop’s success lays the foundation for a broader vision of improving research administration not only within Aga Khan University (AKU) but across institutions in Pakistan. Building upon the workshop’s achievements, several future directions can be considered such as expanding workshop content to cover advanced project management concepts, tailoring training sessions for diverse participant needs, establishing certification courses for continuous learning, and fostering institutional collaboration through shared knowledge events. These initiatives aim to build upon the workshop’s achievements and enhance grant administration practices nationwide, ultimately strengthening the research ecosystem in Pakistan.

## Conclusion

The capacity-strengthening workshop aimed at enhancing grant administration skills in Pakistan yielded promising results, notably evident through pre- and post-survey assessments demonstrating significant improvements in participants’ understanding of grant administration processes, particularly in Islamabad. Key factors contributing to the workshop’s success included its comprehensive content, dynamic teaching methods, and interactive learning environment, bolstered by practical sessions and diverse participant backgrounds fostering cross-learning and networking opportunities. The workshop served as a valuable foundation for novice researchers, instilling confidence in their grant administration abilities. Tailored capacity-strengthening programs are crucial for addressing the unique needs of academic and research institutions, while collaborative learning enhances collective knowledge. The outcomes of this research highlight the effectiveness of tailored capacity-strengthening programs that address the diverse needs of institutions at various stages of development. Institutions like AKU, with established infrastructures, benefited from enhanced capacity, while institutions with less developed systems, such as those in Islamabad, saw gains from foundational capacity building. This dual approach helped create a more cohesive national strategy for improving grant administration across institutions with varying levels of existing support. This research underscores the significance of capacity-strengthening workshops in strengthening grant administration within the academic and research landscape of Pakistan, providing a roadmap for future endeavors to further enhance grant administration capabilities and foster a more robust research ecosystem.

## Supporting information

S1 File Pre-workshop survey questionnaire. (DOCX)

S2 File Detailed workshop content. (DOCX)

S3 File Post-workshop survey questionnaire. (DOCX)

S1 Data set (XLSX)
