# Supplementary material for: Building block for success: A case study of capacity-strengthening in grant administration for Pakistani universities
Source: PLoS One. 2024 Nov 22;19(11):e0314141. doi: 10.1371/journal.pone.0314141 (PMC11584076; doi:10.1371/journal.pone.0314141)
Supplement: S1 File — (DOCX) [file pone.0314141.s001.docx]

# **Supporting Information**

**Annexure A: Pre-workshop questionnaire**

**Pre-workshop Survey**: A mixed-method approach was used to determine the needs and expectations of the participants. An initial step involved designing a pre-workshop questionnaire distributed via Google Forms. The pre-workshop survey included the following questions:

- How much prior experience in Grant Administration and/or submission do you have?
- Please rate your level of knowledge or familiarity with the best practices at each stage on a scale of 1 to 5 [Pre-Award, Award, Post-Award, and Close-Out].
- Please rate your current level of knowledge or familiarity with the following topics on a scale of 1 to 5 [Preparing and submitting the grant proposal, Budgeting and financial management for grants, Essential documents used throughout the grant administration, Monitoring and managing grant-funded activities].
- What are your expectations for this training program?
- How do you prefer to learn? (e.g., hands-on activities, lectures, group discussions, online modules).
- Is there a specific topic you would like the facilitators to focus on during the training?
