# Supplementary material for: Building block for success: A case study of capacity-strengthening in grant administration for Pakistani universities
Source: PLoS One. 2024 Nov 22;19(11):e0314141. doi: 10.1371/journal.pone.0314141 (PMC11584076; doi:10.1371/journal.pone.0314141)
Supplement: S2 File — (DOCX) [file pone.0314141.s002.docx]

# **Supporting Information**

**Annexure B: Detailed Workshop Content**

**Workshop Content**

The workshop content was structured into 10 modules, each designed to cover specific aspects of grants administration:

### **Day 1**

**Overview and importance of Research:** This session provided an introduction to the world of research and its crucial role in solving the most pressing societal issues using rigorous scientific methods.

**Sourcing funding opportunities:** This section focused on where and how to find relevant grant opportunities. Participants were shown how to search for funding opportunities on grant databases, university websites, and government agencies

**Pre-award Management:** Participants learned about the critical steps involved in preparing for a grant application, from identifying opportunities to understanding pre-award processes.

**Stages of Proposal Development:** This module explored the various phases involved in crafting a compelling grant proposal. Participants were introduced to the step-by-step process of creating a proposal, from initial concept development to final submission.

**Budget Preparation:** Participants gained insights into creating effective budgets for grant proposals. They learned how to allocate funds for various project components, providing examples of how different line items are budgeted, including personnel, equipment, and travel costs.

**Approval and Submission Processes:** In this session participants were guided through the internal review and approval processes within their organization, highlighting the importance of adherence to submission deadlines.

**Simulation Game:** The simulation game conducted during Day 1 of the workshop enabled participants to apply the knowledge and skills they had gained during the workshop to a real-world scenario. They were given a Funding Opportunity Announcement (FOA) for an NIH G11 grant. In this exercise, participants had the task of thoroughly examining the FOA to extract vital information such as:

Deadlines: Participants had to identify and note the submission deadlines specified in the FOA. This involved understanding the application submission timeline and ensuring compliance with the submission dates.

Documents Required: The FOA likely outlined the essential documents and forms needed for the grant application. Participants were required to identify and list these documents, gaining insight into the paperwork involved.

Proposal Development: This part of the simulation game focused on recognizing the FOA's requirements and guidelines for proposal development. Participants learned how to align their project ideas with the FOA's objectives.

Submission Procedures: Participants were tasked with understanding the submission process as detailed in the FOA. They learned how to navigate electronic submission systems and what steps to follow when submitting their grant application.

Budget Preparation: The FOA may have contained instructions on creating a budget for the proposed project. Participants practiced identifying these instructions and understanding the budgeting requirements.

**Award Management:** This part delved into what happens after a grant is awarded and the steps involved in effectively managing the award. Moreover, various types of contracts were also discussed, along with their implications in research administration.

### **Day 2**

**Overall Post-award Management:** The focus shifted to post-award responsibilities, including key areas of concentration in this phase. Participants were guided through the essential tasks involved in managing a grant after it has been awarded, from budget tracking to compliance.

**Financial Management in Post-award:** This module covered financial management strategies specific to grant-funded projects, including budget tracking, expense reporting, and financial accountability.

**Roles and responsibilities of PI and GA:** Participants explored the respective roles of principal investigators (PIs) and grant administrators in grant-funded projects.

**Close-out Management:** The close-out phase of grant management was discussed, emphasizing the importance of completing all tasks and reports at the end of the project.

**Ethics in Research:** The module focused on the ethical considerations in research and grant management.

**Project Management Skills:** This session addressed project management skills essential for successful grant administration. For example, participants learned about creating work breakdown structures (WBS) and risk management plans to ensure smooth project execution.

**Career Development in Research Administration:** The final module explored career development opportunities in the field of research administration.
