# Supplementary material for: Building block for success: A case study of capacity-strengthening in grant administration for Pakistani universities
Source: PLoS One. 2024 Nov 22;19(11):e0314141. doi: 10.1371/journal.pone.0314141 (PMC11584076; doi:10.1371/journal.pone.0314141)
Supplement: S3 File — (DOCX) [file pone.0314141.s003.docx]

# **Supporting Information**

**Annexure C: Post-workshop questionnaire**

The post-survey included the following questions:

- Please rate the following aspects of the training on a scale of 1 to 5, with 1 being "Strongly Disagree" and 5 being "Strongly Agree." [The content of the training was relevant to my role].
- The training materials were well-organized and easy to understand.
- The training sessions were engaging and interactive.
- The trainer was knowledgeable and able to address questions effectively.
- The training duration was appropriate for covering the material.
- How confident do you feel about the best practices for each stage after the training, on a scale of 1 to 5 [Pre-Award, Post-Award, Award, Close-Out].
- How confident do you feel about the following topics after completing the training? Please rate on a scale of 1 to 5 [Preparing and submitting the grant proposal, Budgeting and financial management for grants, Essential documents used throughout the grant administration process, Monitoring and managing grant-funded activities].
- How do you expect the knowledge and skills gained from this training to impact your role?
- Do you have any suggestions for improving future grant administration training sessions?
